# GLP‐1 Medicines in Perspective: Approaches to Optimize Outcome

**DOI:** 10.1111/1753-0407.70264

**Published:** 2026-08-19

**Authors:** Zachary Bloomgarden

**Affiliations:** ^1^ Division of Endocrinology, Diabetes and Bone Disease, Department of Medicine Icahn School of Medicine at Mount Sinai New York New York USA

The recent history of glucagon‐like peptide‐1 (GLP‐1)‐based therapy has been told largely as a succession of efficacy milestones: greater HbA1c lowering, greater weight loss, and progressively broader cardiovascular and kidney benefits. The newest literature suggests that this is an incomplete description. The clinically important question is no longer, “How much weight can be lost?” A more complex set of questions must be asked, pertaining to which biological effects matter for a particular patient, how durable are they, and what should be combined with GLP‐1 receptor activation (RA) to improve the quality rather than simply the magnitude of response?

## 1 New Information About Clinical Use

Two developments could materially alter how GLP‐1 therapies are delivered. First, semaglutide was recently reformulated to require a lower oral dose for the same effect [1], and another agent, orfoglipron, has been brought to market in the US [2]. Orfoglipron, along with aleniglipron and elecoglipron, allows oral dosing of nonpeptide small‐molecule GLP‐1RA with clinically meaningful weight and glucose lowering [3, 4]. The small molecule GLP‐1RAs can be taken without the food, water, and post‐dose restrictions required for oral semaglutide, and all the agents are associated with appreciable placebo‐adjusted weight loss. Oral treatment, particularly using small molecules, could reduce barriers related to injections, refrigeration, manufacturing complexity, and supply.

Second, chronic therapy is beginning to be studied in terms of both induction and maintenance of treatment rather than as an indefinite fixed‐dose prescription. In SURMOUNT‐MAINTAIN, participants who continued maximum‐tolerated tirzepatide after initial weight loss essentially maintained their weight, whereas withdrawal produced substantial regain. De‐escalation to 5 mg preserved a meaningful, although incomplete, portion of the benefit [5]. This raises the clinically important possibility that after effective induction some patients may be managed with lower doses, preserving the desired metabolic and functional result. A recent review emphasizes that cost, access, gastrointestinal intolerance, and perceived lack of benefit commonly lead to treatment interruption, followed by weight regain and deterioration of glycemic and cardiometabolic markers [6]. Persistence and affordability are important aspects of therapeutic efficacy, not merely implementation details.

## 2 Novel Concepts of Mechanism: Effects Beyond Weight Loss

A number of studies challenge the simple hypothesis suggesting that GLP‐1RA causes weight loss, which in turn explains all subsequent benefit.

Consider the SYNCHRONIZE trial of the oxyntomodulin analog survodutide, which activates both the glucagon receptor and the GLP‐1 receptor. Although the agent was associated with a lower mean change in body weight than that expected with the class, changing at Week 76 by just −12.2%, it led to a 63.1% reduction in liver fat, considerably greater than the 27.8% and 34.0% reductions in total body fat and visceral fat, respectively [7]. In STRIDE, semaglutide improved maximum and pain‐free walking distance in people with Type 2 diabetes and symptomatic peripheral artery disease despite substantially less weight loss than seen in obesity trials [8]. A pooled analysis of SELECT, FLOW, and SOUL similarly found reduced kidney events across populations and across strata of BMI, glycemia, kidney function, and albuminuria [9]. None of these observations prove weight independence, but all make a purely weight‐mediated explanation less likely.

In an experimental model of steatohepatitis, semaglutide acted through GLP‐1 receptors in hepatic sinusoidal endothelial cells, with these cells in turn appearing to function as signaling hubs coordinating inflammatory and intercellular responses [10]. The important concept is that a relatively restricted receptor population can reorganize organ physiology through cell‐to‐cell signaling rather than through direct receptor expression on every target cell. An analogous mechanism may apply in the brain. A blood–brain‐barrier‐penetrant oral GLP‐1 receptor agonist, OHP2, activated astrocytic GLP‐1 receptors in Alzheimer disease models, increasing glycolysis and lactate release, neuronal histone lactylation, and neuron‐astrocyte lipid transfer [11]. Although not providing evidence for treating Alzheimer's disease, the notion of a lactate‐histone‐lipid axis is intriguing, and would provide a similar mechanism by which neurons not expressing the GLP‐1R might respond to GLP‐1RA administration.

Together, these studies support the notion that GLP‐1 medicines may function as inter‐organ and intercellular signaling modifiers, with effects that need not scale linearly with weight loss. The dose needed for organ protection may therefore not always be the dose that maximizes weight reduction.

## 3 New Information About Beneficial Effects

The evidence for benefit is expanding in a number of domains. STRIDE makes physical function in symptomatic vascular disease a GLP‐1 outcome, not merely prevention of myocardial infarction or stroke [8]. The SELECT/FLOW/SOUL analysis strengthens the case for kidney protection across a broad cardiometabolic spectrum [9].

Other findings are intriguing but less definitive. In a Swedish national cohort of people with depression or anxiety, semaglutide use was associated with lower rates of worsening mental illness, including depression, anxiety, substance‐use outcomes, and self‐harm [12]. These observational associations do not establish a psychiatric indication, but they are directionally consistent with emerging evidence that GLP‐1 pathways influence reward and motivational circuitry. The use of GLP‐1RAs in Type 1 diabetes is another developing area. In a 24‐person Phase 2 trial, tirzepatide produced large weight loss and substantial reductions in insulin requirements over 12 weeks [13]. This is important proof‐of‐concept for the increasingly common Type 1 diabetes phenotype characterized by obesity and insulin resistance. However, a small short‐term trial cannot establish safety with respect to diabetic ketoacidosis, severe hypoglycemia, nutritional compromise, or long‐term complications.

## 4 New Information About Potential Adverse Effects

Gastrointestinal adverse events remain the most frequent limitation of GLP‐1 and multi‐agonist therapy, but recent studies suggest additional potential issues. A target‐trial emulation found a modestly higher risk of alopecia among people starting GLP‐1RA than among SGLT2‐ or DPP‐4‐inhibitor initiators [14]. Rapid weight loss, nutritional change, and telogen effluvium are plausible alternatives to direct follicular toxicity. Although not medically dangerous, this may be highly important to patients and affect persistence.

Body composition requires even more nuance. A meta‐analysis estimated that lean tissue accounts for roughly one‐quarter to one‐third of weight lost with GLP‐1RA [15]. Fat‐free mass, however, is not synonymous with skeletal muscle, and loss of DEXA lean mass is not equivalent to sarcopenia. Concern is greatest in older adults with low physiologic reserve, for whom weight reduction may worsen frailty or produce muscle loss that is difficult to recover [16].

A novel strategy was studied in the EMBRAZE Phase 2 trial, in which the myostatin inhibitor apitegromab was administered in combination with tirzepatide, leading to preservation of approximately 55% of the lean mass otherwise lost, without reducing total weight loss [17]. Whether this translates into preserved strength or function remains unknown. As with the survodutide trial, weight loss composition rather than its absolute magnitude emerges as a new therapeutic target.

Behavioral and nutritional safety also deserves attention. An NEJM Perspective warns that powerful appetite suppression could aggravate restrictive eating or facilitate misuse in people with eating disorders, while possible benefit for binge‐type behaviors remains incompletely studied [18]. Finally, two cases of choroidal lymphoid hyperplasia that resolved after dulaglutide withdrawal represent an unusual pharmacovigilance signal [19]. Two cases cannot establish causality or a class effect, but with growing GLP‐1RA use the recognition of rare events becomes increasingly important.

## 5 Comparison With Other Medicines and Rational Combinations

The next phase of metabolic pharmacology may be defined less by competition among single agents than by complementary mechanisms. Cagrilintide‐semaglutide provides one example. In REIMAGINE 2, adding amylin‐receptor agonism to semaglutide produced only a modest incremental HbA1c effect but a much larger incremental weight effect [20]. This suggests that the added pathway becomes most valuable where appetite and weight remain therapeutic targets after GLP‐1 glucose effects are already near a ceiling.

Retatrutide shows a different aspect of the notion of combination treatment approaches. By adding glucagon receptor agonism to GIP and GLP‐1 signaling, it may add energy expenditure and lipid oxidation while incretin activity restrains glucagon‐related hyperglycemia. Phase 3 data show very large weight loss with robust HbA1c lowering, although the absence of an active comparator leaves unanswered whether triple agonism provides enough incremental benefit over current dual agonism to offset the greater gastrointestinal burden reported with this agent [21]. More receptors do not automatically mean greater net clinical value.

GLP‐1RA and SGLT2 inhibitors may be the most attractive complementary combination, with substantial difference in mechanisms of action: SGLT2 inhibition alters renal energy expenditure, hemodynamics, and natriuresis, as well as being associated with improvement in heart failure, while GLP‐1 therapy more strongly affects weight, appetite, atherosclerotic outcomes, and potentially tissue‐specific inflammatory signaling. A recent review finds greater reductions in HbA1c, weight, and blood pressure when the classes are combined, and raises the potential of additive organ protection, recognizing that randomized hard‐outcome evidence specifically testing the combination remains limited [22].

Bariatric surgery remains an important comparator. In a real‐world study of people with obesity and Type 2 diabetes, sleeve gastrectomy produced the highest 1‐year probability of achieving both at least 20% weight loss and HbA1c below 5.7%, followed by tirzepatide and semaglutide [23]. Yet treatment selection was nonrandom and the time courses differ: surgery reaches maximal loss earlier, whereas incretin therapy may continue to work beyond 1 year. Long‐term outcome studies, although difficult to design, are likely to be required to fully compare these options; lacking this we will need to rely on clinical judgment in the foreseeable future.

## 6 From Maximal Weight Loss to Optimal Treatment

Across these studies, a unifying principle emerges. The goal should not be to maximize weight loss in every patient. It should be to optimize across multiple dimensions of therapeutic outcome: glycemia, dyslipidemia, cardiovascular and kidney risk, liver disease, physical function, body composition, tolerability, treatment burden, affordability, and patient priorities.

This distinction becomes more important as drugs become more potent. In a younger patient with severe obesity, weight loss of more than 20% is likely to be the most desirable. In an older person at risk for frailty, the appropriate strategy may be slower loss, resistance exercise and nutritional support, dose de‐escalation after induction, or maintenance without further weight reduction. In chronic kidney disease or heart failure, adding an SGLT2 inhibitor may be more valuable than intensifying incretin‐mediated weight loss. With hepatic steatosis, greater reduction in liver fat may be more important than the absolute magnitude of weight loss. With excessive lean mass loss, combination strategies of effective incretin therapy with muscle‐directed treatment may be optimal.

## 7 Conclusion

GLP‐1‐based medicines have evolving from glucose‐lowering anti‐obesity drugs to a therapeutic platform with multiple potential actions. Oral small molecules may broaden access; amylin, GIP, and glucagon combinations may extend efficacy; and basic mechanistic studies along with clinical trials suggest vascular, renal, hepatic, and neural actions that cannot be explained solely by body‐weight change. At the same time, withdrawal, lean‐mass loss, behavioral vulnerability, tolerability, cost, and rare adverse signals become more important. We must therefore ask, “Which mechanism, dose, duration, and combination produce the most desirable (and durable) clinical outcome for a given patient?” Our goal is not the lowest body weight achieved, but sustained organ protection, preserved function, acceptable burden, and long‐term health.

## Funding

The author has nothing to report.

## Conflicts of Interest

The author declares no conflicts of interest.
